# Supplementary figures and images for: Soluble chitosan derivative treats wound infections and promotes wound healing in a novel MRSA-infected porcine partial-thickness burn wound model
Source: PLoS One. 2022 Oct 14;17(10):e0274455. doi: 10.1371/journal.pone.0274455 (PMC9565743; doi:10.1371/journal.pone.0274455)

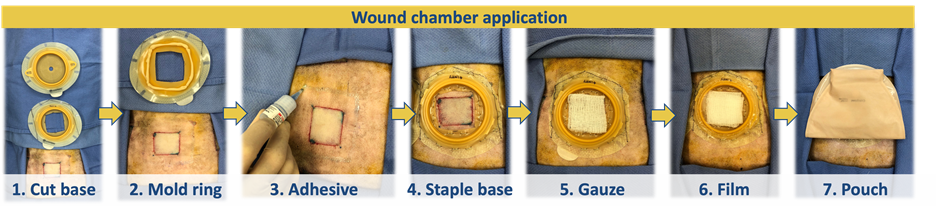

Supplement: S1 Fig — Wound chambers were engineered as a two-piece structure (base and cover) to surround, isolate, and cover individual porcine wounds. (TIF) [file pone.0274455.s001.tif]

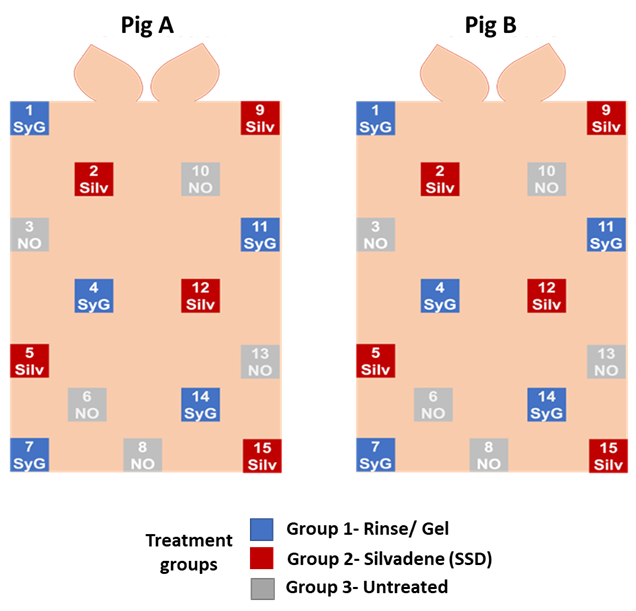

Supplement: S2 Fig — (TIF) [file pone.0274455.s002.tif]

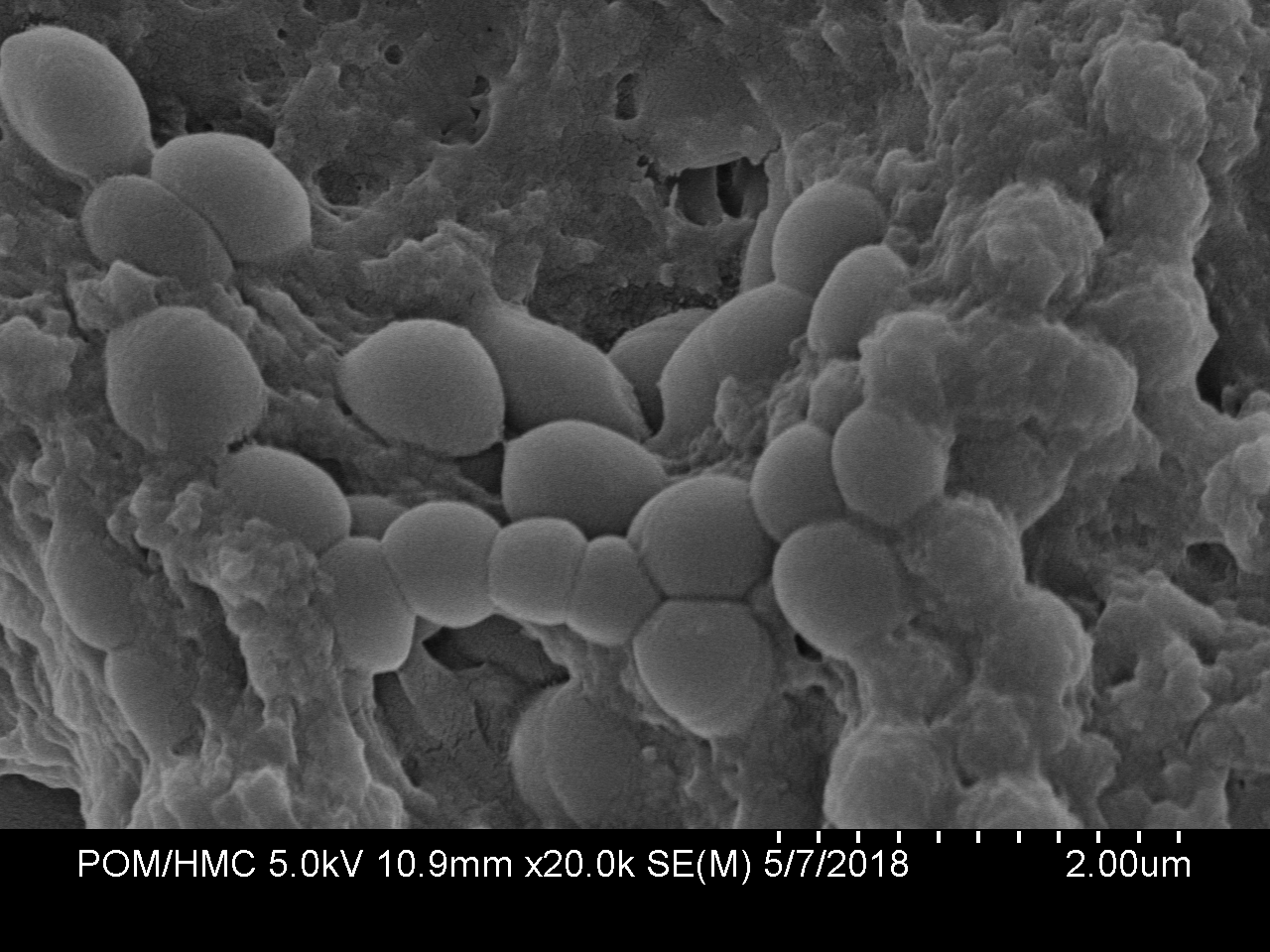

Supplement: S3 Fig — (TIF) [file pone.0274455.s003.tif]
