# Supplementary material for: Soluble chitosan derivative treats wound infections and promotes wound healing in a novel MRSA-infected porcine partial-thickness burn wound model
Source: PLoS One. 2022 Oct 14;17(10):e0274455. doi: 10.1371/journal.pone.0274455 (PMC9565743; doi:10.1371/journal.pone.0274455)
Supplement: S1 File — (PDF) [file pone.0274455.s004.pdf]

		0	3	5	7
	WoundCode	1/5/2018	1/8/2018	1/10/2018	1/12/2018
**NEGATIVE CONTROL**	AHN1	16.0	16.0	15.1	14.7
	AHN2	16.0	15.6	15.0	14.8
	AHN3	16.0	15.7	14.7	13.6
	**AHN4**	16.0	15.3	14.4	13.9
	AHN5	16.0	15.4	14.9	14.4
	AHN6	16.0	15.9	14.0	13.2
	AHN7	16.0	15.7	15.0	15.1
	AHN8	16.0	15.3	14.8	14.5
	**AHN9**	16.0	15.8	14.7	13.7
	AHN10	16.0	15.5	15.1	14.8
	AHN11	16.0	15.3	15.2	14.5
	AHN12	16.0	15.8	15.6	14.7
	AHN13	16.0	15.6	14.6	12.5
	AHN14	16.0	15.5	14.9	13.8
	AHN15	16.0	15.8	15.2	14.2

		**0**	**3**	**5**	**7**
			**2/19/2018**	**2/21/2018**	**2/23/2018**
**NO TREATMENT**	BMN3	X	16.2	15.6	14.7
	BMN6	X	20.9	19.9	18.6
	BMN8	X	21.8	20.1	18.0
	BMN10	X	18.1	17.8	16.9
	BMN13	X	18.3	17.0	16.1
**SILVADENE**	BMS2	X	17.9	16.3	15.5
	BMS5	X	17.7	17.6	16.0
	BMS9	X	19.5	18.4	16.3
	BMS12	X	16.4	15.6	14.1
	BMS15	X	16.1	15.8	14.0
**SYN01**	BMG1	X	17.9	16.8	11.8
	BMG4	X	16.6	16.8	12.1
	BMG7	X	20.2	18.7	11.7
	BMG11	X	16.7	16.6	13.2
	BMG14	X	19.3	17.5	14.7

		**0**	**3**	**5**	**7**
**MEAN CM2**	HN	16.0	15.6	14.9	14.2
TOTAL WOUI	MN		19.1	18.1	16.9
	MS		17.5	16.7	15.2
	MG		18.1	17.3	12.7

SD	HN	0.0	0.22635833	0.37834225	0.70589356
	MN		2.26781833	1.93494413	1.56511661
	MS		1.36371251	1.22164467	1.06249762
	MG		1.58584968	0.86481859	1.27208989
SE	HN	0.0	0.1	0.1	0.2
	MN		1.0	0.9	0.7
	MS		0.6	0.5	0.5
	MG		0.7	0.4	0.6
		**0**	**3**	**5**	**7**
% total woun	HN	0	0	5	9
	MN	#DIV/0!	0	5	11
	MS	#DIV/0!	0	5	13
	MG	#DIV/0!	0	5	30

% Tot Wound Closure

11	13	17	19	21	24	26
1/16/2018	1/18/2018	1/22/2018	1/24/2018	1/26/2018	1/29/2018	1/31/2018
14.3	5.0	2.0	0.0	0.0	0.0	0.0
14.7	8.0	7.0	3.0	2.8	1.3	0.4
9.3	6.2	6.8	0.0	0.0	0.0	0.0
13.9	11.9	8.5	5.5	2.1	0.4	0.2
14.1	10.3	10.1	6.4	6.0	3.3	0.0
13.3	10.1	4.4	4.3	1.0	0.6	0.0
14.8	8.1	7.2	4.0	4.0	0.3	0.0
14.3	9.9	4.5	2.8	2.3	0.9	0.0
13.4	7.5	5.3	0.9	2.8	3.4	1.6
13.7	6.2	0.9	1.1	0.0	0.0	0.0
14.2	5.8	1.4	0.0	0.0	0.0	0.0
11.2	2.4	1.5	1.2	0.0	0.0	0.0
12.6	9.4	1.8	0.7	0.7	0.6	0.0
13.7	10.8	5.0	1.6	0.0	0.0	0.0
13.2	7.5	5.0	2.7	0.9	0.0	0.0

**11**	**13**	**17**	**19**	**21**	**24**	**26**
**2/26/2018**	**2/28/2018**	**3/2/2018**	**3/6/2018**	**3/8/2018**	**3/12/2018**	**3/14/2018**
13.6	6.7	3.0	4.2	3.3	0.0	0.0
12.9	10.1	8.8	3.3	1.9	0.9	0.0
11.6	9.6	3.5	2.5	2.3	1.3	1.3
13.3	13.1	5.7	2.8	0.8	0.3	0.3
13.7	7.2	6.8	3.0	1.6	0.5	0.5
12.1	6.0	1.6	0.0	0.0	0.0	0.0
13.3	5.7	0.9	0.3	0.0	0.0	0.0
12.0	7.6	6.1	4.0	1.7	0.0	0.0
10.1	4.6	2.0	1.0	0.8	0.0	0.0
13.1	4.9	0.4	0.1	0.0	0.0	0.0
10.4	3.9	3.6	1.2	0.0	0.0	0.0
8.4	0.5	0.0	0.0	0.0	0.0	0.0
2.8	0.4	0.0	0.0	0.0	0.0	0.0
9.0	7.8	4.8	1.3	0.0	0.0	0.0
3.4	0.0	0.0	0.0	0.0	0.0	0.0

**11**	**13**	**17**	**19**	**21**	**24**	**26**
13.4	7.9	4.8	2.3	1.5	0.7	0.1
13.0	9.3	5.6	3.1	2.0	0.6	0.4
12.1	5.8	2.2	1.1	0.5	0.0	0.0
6.8	2.5	1.7	0.5	0.0	0.0	0.0

1.44330969	2.54187782	2.82129656	2.04177794	1.79816445	1.13841996	0.41724721
0.85417153	2.54990876	2.36912701	0.64902596	0.93045381	0.50692682	0.54370185
1.2740923	1.18332468	2.26285092	1.67195954	0.73576627	0	0
3.45269204	3.34857843	2.36101817	0.69648058	0	0	0

0.4	0.7	0.7	0.5	0.5	0.3	0.1
0.4	1.1	1.1	0.3	0.4	0.2	0.2
0.6	0.5	1.0	0.7	0.3	0.0	0.0
1.5	1.5	1.1	0.3	0.0	0.0	0.0

**11**	**13**	**17**	**19**	**21**	**24**	**26**
14	49	70	85	90	95	99
32	51	71	83	90	97	98
31	67	87	94	97	100	100
62	86	91	97	100	100	100

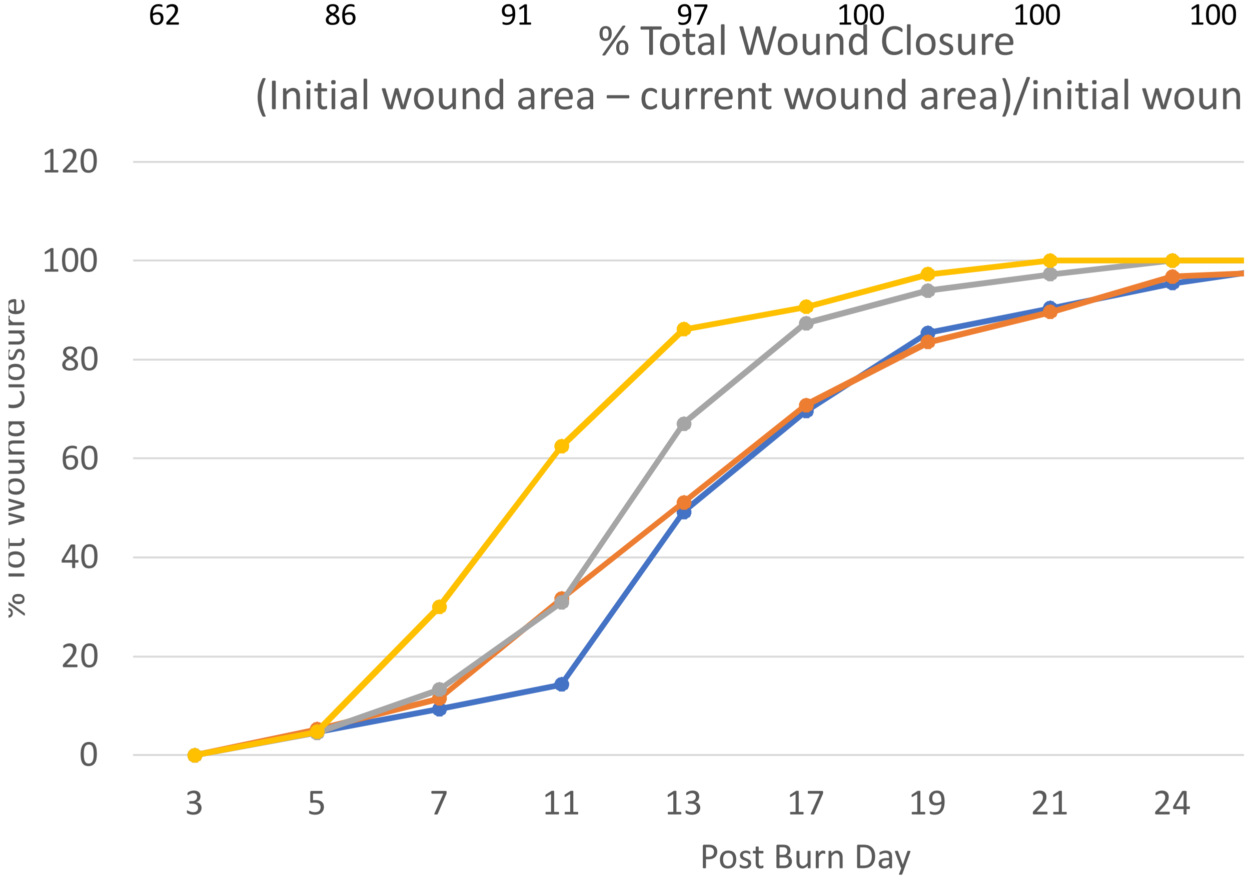

28
2/2/2018
0.0
0.0
0.0
0.0
0.0
0.0
0.0
0.0
0.0
0.0
0.0
0.0
0.0
0.0
0.0

**28**
**3/16/2018**
0.0
0.0
0.8
0.0
0.0
0.0
0.0
0.0
0.0
0.0
0.0
0.0
0.0
0.0
0.0

**28**
0.0
0.2
0.0
0.0

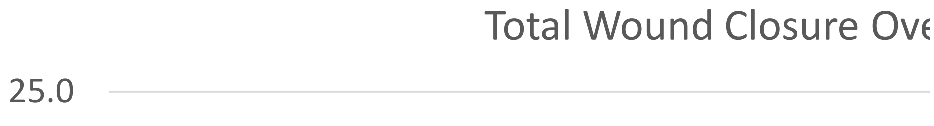

0
0.34032955
0
0

0.0
0.2
0.0
0.0

28
100
99
100
100

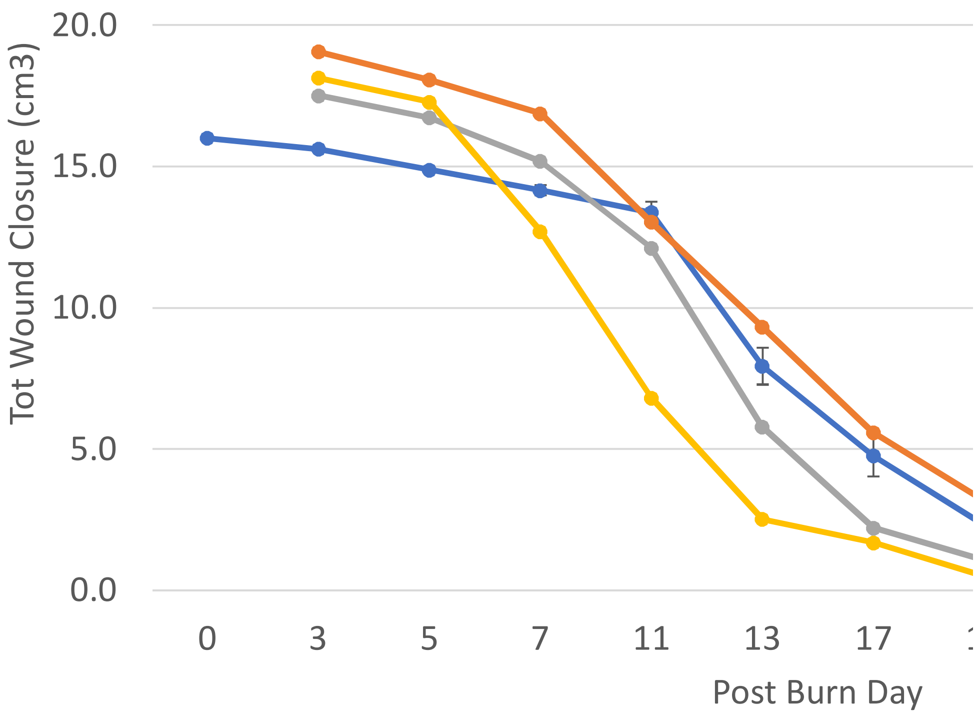

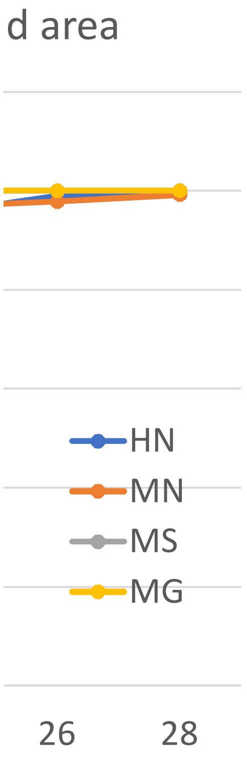

er Time

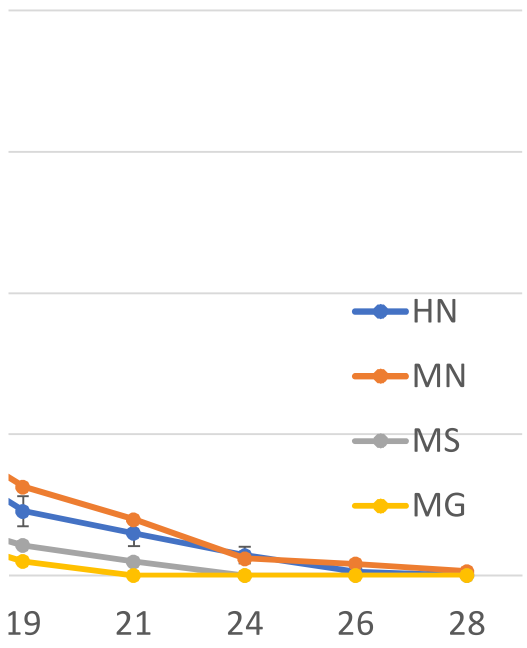
HN
MN
MS
MG
19
21
24
26
28

Sample	CFU TSAB (S. aureus)	Dilution	CFU TSAB (P. aeruginosa)	Dilution	CFU Mac.	Dilution
BMN3	155	10000			0	0
BMS5	106	100			0	0
BMS9	164	10000			0	0
BMG11	119	100			0	0
BMN13	173	1000			0	0

Weight Tissue (g)	CFU/g (S. aureus)	CFU/g (P. aeruginosa) TSAB	CFU/g (P. aeruginosa) MAC
0.07	221428571.4	#DIV/0!	#DIV/0!
0.0857	1236872.812	#DIV/0!	#DIV/0!
0.0946	173361522.2	#DIV/0!	#DIV/0!
0.0514	2315175.097	#DIV/0!	#DIV/0!
0.0852	20305164.32	#DIV/0!	#DIV/0!

Sample	CFU TSAB (S. aureus)	Dilution	CFU TSAB (P. aeruginosa)	Dilution	CFU Mac.	Dilution
BMG1	35	10			0	0
BMN3	47	1000			0	0
BMS5	187	100			0	0
BMS9	115	10000			0	0
BMG11	170	100			0	0
BMN13	96	1000			0	0

Weight Tissue (g)	CFU/g (S. aureus)	CFU/g (P. aeruginosa) TSAB	CFU/g (P. aeruginosa) MAC
0.0269	130111.5242	#DIV/0!	#DIV/0!
0.0556	8453237.41	#DIV/0!	#DIV/0!
0.0403	4640198.511	#DIV/0!	#DIV/0!
0.0609	188834154.4	#DIV/0!	#DIV/0!
0.0451	3769401.33	#DIV/0!	#DIV/0!
0.037	25945945.95	#DIV/0!	#DIV/0!

Sample	CFU TSAB (S. aureus)	Dilution	CFU TSAB (P. aeruginosa)	Dilution	CFU Mac.	Dilution
BMG1	129	10			0	0
BMN3	229	100			0	0
BMS5	306	100			0	0
BMS9	55	10000			0	0
BMG11	65	10			0	0
BMN13	283	10			0	0

Weight Tissue (g)	CFU/g (S. aureus)	CFU/g (P. aeruginosa) TSAB	CFU/g (P. aeruginosa) MAC
0.0433	297921.4781	#DIV/0!	#DIV/0!
0.0444	5157657.658	#DIV/0!	#DIV/0!
0.0319	9592476.489	#DIV/0!	#DIV/0!
0.0454	121145374.4	#DIV/0!	#DIV/0!
0.0345	188405.7971	#DIV/0!	#DIV/0!
0.0291	972508.5911	#DIV/0!	#DIV/0!

Sample	CFU TSAB (S. aureus)	Dilution	CFU TSAB (P. aeruginosa)	Dilution	CFU Mac.	Dilution
BMG1	125	100			0	0
BMN3	100	10000			0	0
BMS5	183	1000			0	0
BMS9	175	100			0	0
BMG11	52	10000			0	0
BMN13	142	100			0	0

Weight Tissue (g)	CFU/g (S. aureus)	CFU/g (P. aeruginosa) TSAB	CFU/g (P. aeruginosa) MAC
0.0598	2090301.003	#DIV/0!	#DIV/0!
0.0857	116686114.4	#DIV/0!	#DIV/0!
0.078	23461538.46	#DIV/0!	#DIV/0!
0.071	2464788.732	#DIV/0!	#DIV/0!
0.0855	60818713.45	#DIV/0!	#DIV/0!
0.0482	2946058.091	#DIV/0!	#DIV/0!

Sample	CFU TSAB (S. aureus)	Dilution	CFU TSAB (P. aeruginosa)	Dilution	CFU Mac.	Dilution
BMG1	57	1000			0	0
BMN3	115	10000			0	0
BMS5	176	100			0	0
BMS9	53	10000			0	0
BMG11	36	10000			0	0
BMN13	55	10000			0	0

Weight Tissue (g)	CFU/g (S. aureus)	CFU/g (P. aeruginosa) TSAB	CFU/g (P. aeruginosa) MAC
0.0496	11491935.48	#DIV/0!	#DIV/0!
0.0283	406360424	#DIV/0!	#DIV/0!
0.0454	3876651.982	#DIV/0!	#DIV/0!
0.0465	113978494.6	#DIV/0!	#DIV/0!
0.044	81818181.82	#DIV/0!	#DIV/0!
0.0309	177993527.5	#DIV/0!	#DIV/0!

Sample	CFU TSAB (S. aureus)	Dilution	CFU TSAB (P. aeruginosa)	Dilution	CFU Mac.	Dilution
BMG1	419	1000			0	0
BMN3	443	1000			0	0
BMS5	440	1000			0	0
BMS9	586	1000			0	0
BMG11	590	1000			0	0
BMN13	605	1000			0	0

Weight Tissue (g)	CFU/g (S. aureus)	CFU/g (P. aeruginosa) TSAB	CFU/g (P. aeruginosa) MAC
0.0411	101946472	#DIV/0!	#DIV/0!
0.031	142903225.8	#DIV/0!	#DIV/0!
0.0338	130177514.8	#DIV/0!	#DIV/0!
0.0469	124946695.1	#DIV/0!	#DIV/0!
0.0339	174041297.9	#DIV/0!	#DIV/0!
0.0388	155927835.1	#DIV/0!	#DIV/0!

Synepure

	TSB	1X PBS	1	3	5	10
	250000000	150000000	200000	30000	200	200
	190000000	160000000	500000	40000	300	100
	170000000	140000000	300000	20000	200	300
	160000000	190000000	900000	50000	200	200
	170000000	150000000	500000	30000	300	200
	180000000	170000000	700000	40000	200	100
	160000000	170000000	600000	50000	300	200
	140000000	180000000	700000	70000	400	500
	140000000	160000000	600000	90000	200	400
Avg	1.73E+08	1.63E+08	5.56E+05	4.67E+04	2.56E+02	2.44E+02
std dev	3.04E+07	1.45E+07	1.95E+05	2.00E+04	6.67E+01	1.22E+02

	TSB			
	250000000	160000000	190000000	160000000
	190000000	170000000	150000000	170000000
	170000000	150000000	170000000	160000000
**Avg**	**2.03E+08**	**1.60E+08**	**1.70E+08**	**1.63E+08**
Stdev	33993463	8164965.8	16329932	4714045.2

	1X PBS			
	150000000	170000000	140000000	150000000
	160000000	150000000	160000000	170000000
	140000000	180000000	180000000	190000000
**Avg**	**1.50E+08**	**1.67E+08**	**1.60E+08**	**1.70E+08**
Stdev	8164965.8	12472191	16329932	16329932

Catasyn

	TSB	1X PBS	10	30	60	24hr
	130000000	130000000	15000	1300	0	0
	140000000	120000000	14000	1400	0	0
	170000000	150000000	15000	1500	0	0
	160000000	160000000	14000	1400	0	0
	170000000	170000000	12000	1500	0	0
	130000000	150000000	16000	1400	0	0
	130000000	130000000	11000	1400	0	0
	140000000	160000000	10000	1300	0	0
	160000000	170000000	12000	1300	0	0
Avg	1.48E+08	1.49E+08	1.32E+04	1.39E+03	0.00E+00	0.00E+00
std dev	1.57E+07	1.68E+07	1.88E+03	7.17E+01	0.00E+00	0.00E+00

TSB			
130000000	120000000	170000000	160000000

	140000000	150000000	190000000	140000000
	170000000	140000000	180000000	170000000
AV	**1.47E+08**	**1.37E+08**	**1.80E+08**	**1.57E+08**
STDEv	18618987	13662601	8944271.9	13662601
	1X PBS			
	130000000	120000000	160000000	160000000
	120000000	110000000	150000000	140000000
	150000000	130000000	170000000	170000000
AV	**1.33E+08**	**1.20E+08**	**1.60E+08**	**1.57E+08**
STDEv	13662601	8944271.9	8944271.9	13662601

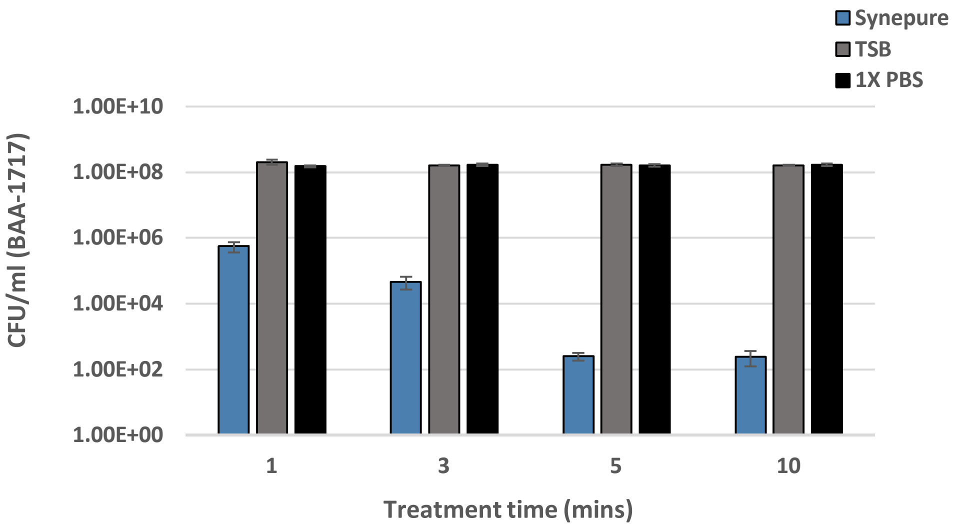
Synepure
TSB
1X PBS
1.00E+10
1.00E+08
1.00E+06
1.00E+04
1.00E+02
1.00E+00
CFU/ml (BAA-1717)
1
3
5
10
Treatment time (mins)

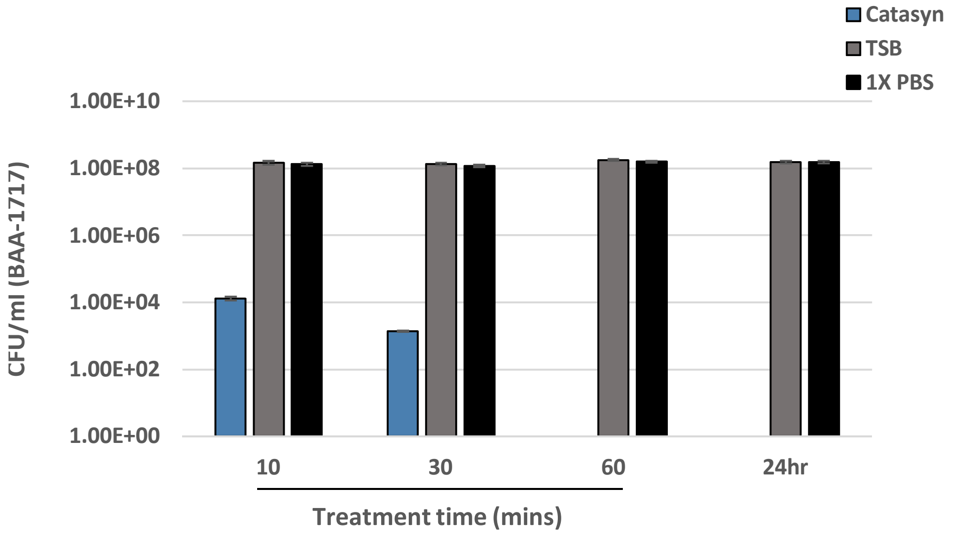
Catasyn
TSB
1X PBS
1.00E+10
1.00E+08
1.00E+06
1.00E+04
1.00E+02
1.00E+00
CFU/ml (BAA-1717)
10
30
60
24hr
Treatment time (mins)

Dr. Rubin Paper- Additional data
Synepure (Lot: 1701-19-028, Exp: 05/29/2022)
Date : 7/1/2019 Treatment time: 1 min

TSB	1X PBS	Synepure
25	15	2
19	16	5
17	14	3
5	5	3
5	5	3
5	5	3
10	10	10
10	10	10
10	10	10
10	10	10
10	10	10
10	10	10

	TSB	1X PBS	Synepure	
	2.50E+08	1.50E+08	2.00E+05	
	1.90E+08	1.60E+08	5.00E+05	
	1.70E+08	1.40E+08	3.00E+05	
**Avg**	**2.03E+08**	**1.50E+08**	**3.33E+05**	**Avg**
STd dev				STd dev

	TSB	
	250000000	1.6E+08
	190000000	1.7E+08
	170000000	1.5E+08
**Avg**	**2.03E+08**	**1.60E+08**
Stdev	33993463.4	8164966

	1X PBS	
	150000000	1.7E+08
	160000000	1.5E+08
	140000000	1.8E+08
**Avg**	**1.50E+08**	**1.67E+08**
Stdev	8164965.81	12472191

Treatment time: 3 min

TSB	1X PBS	Synepure
16	17	3
17	15	4
15	18	2
5	5	2
5	5	2
5	5	2
10	10	10
10	10	10
10	10	10
10	10	10
10	10	10
10	10	10

Treatment time: 5 min

TSB	1X PBS	Synepure
19	14	2
15	16	3
17	18	2
5	5	0
5	5	0
5	5	0
10	10	10
10	10	10
10	10	10
10	10	10
10	10	10
10	10	10

Untreated	1X PBS	Catasyn	
1.60E+08	1.70E+08	3.00E+04	
1.70E+08	1.50E+08	4.00E+04	
1.50E+08	1.80E+08	2.00E+04	
**1.60E+08**	**1.67E+08**	**3.00E+04**	**Avg**
			STd dev

Untreated	1X PBS	Catasyn	
1.90E+08	1.40E+08	2.00E+02	
1.50E+08	1.60E+08	3.00E+02	
1.70E+08	1.80E+08	2.00E+02	
**1.70E+08**	**1.60E+08**	**2.33E+02**	**Avg**
			STd dev

1.9E+08	1.6E+08
1.5E+08	1.7E+08
1.7E+08	1.6E+08
**1.70E+08**	**1.63E+08**
16329932	4714045

1.4E+08	1.5E+08
1.6E+08	1.7E+08
1.8E+08	1.9E+08
**1.60E+08**	**1.70E+08**
16329932	16329932

Treatment time: 10 min

TSB	1X PBS	Synepure
16	15	2
17	17	1
16	19	3
5	5	0
5	5	0
5	5	0
10	10	10
10	10	10
10	10	10
10	10	10
10	10	10
10	10	10

Untreated	1X PBS	Catasyn
1.60E+08	1.50E+08	2.00E+02
1.70E+08	1.70E+08	1.00E+02
1.60E+08	1.90E+08	3.00E+02
**1.63E+08**	**1.70E+08**	**2.00E+02**

Dr. Rubin Paper- Additional data
Synepure (Lot: 1701-19-028, Exp: 05/29/2022)

Date : 7/1/2019

Expreimental replicate 2

	TSB	1X PBS	Synepure	Treatment TSB
Treatment time: 1 min				
	16	19	9	15
	17	15	5	17
	18	17	7	19
	5	5	3	5
	5	5	3	5
	5	5	3	5
	10	10	10	10
	10	10	10	10
	10	10	10	10
	10	10	10	10
	10	10	10	10
	10	10	10	10

	**TSB**	**1X PBS**	**Synepure**		**Untreated**
	1.60E+08	1.90E+08	9.00E+05		1.50E+08
	1.70E+08	1.50E+08	5.00E+05		1.70E+08
	1.80E+08	1.70E+08	7.00E+05		1.90E+08
**Avg**	**1.70E+08**	**1.70E+08**	**7.00E+05**	**Avg**	**1.70E+08**
STd dev				STd dev	

time: 3 min			Treatment time: 5 min				Treatment
**1X PBS**	**Synepure**		**TSB**	**1X PBS**	**Synepure**		**TSB**
17	5		16	14	2		16
18	3		17	14	3		15
16	4		16	17	2		17
5	2		5	5	0		5
5	2		5	5	0		5
5	2		5	5	0		5
10	10		10	10	10		10
10	10		10	10	10		10
10	10		10	10	10		10
10	10		10	10	10		10
10	10		10	10	10		10
10	10		10	10	10		10

**1X PBS**	**Catasyn**		**Untreated**	**1X PBS**	**Catasyn**		**Untreated**
1.70E+08	5.00E+04		1.60E+08	1.40E+08	2.00E+02		1.60E+08
1.80E+08	3.00E+04		1.70E+08	1.40E+08	3.00E+02		1.50E+08
1.60E+08	4.00E+04		1.60E+08	1.70E+08	2.00E+02		1.70E+08
**1.70E+08**	**4.00E+04**	**Avg**	**1.63E+08**	**1.50E+08**	**2.33E+02**	**Avg**	**1.60E+08**
		STd dev				STd dev	

time: 10 min

1X PBS	Synepure
17	2
15	2
18	1
5	0
5	0
5	0
10	10
10	10
10	10
10	10
10	10
10	10

1X PBS	Catasyn
1.70E+08	2.00E+02
1.50E+08	2.00E+02
1.80E+08	1.00E+02
**1.67E+08**	**1.67E+02**

Dr. Rubin Paper- Additional data
Synepure (Lot: 1701-19-028, Exp: 05/29/2022)

Date : 7/1/2019 Treatment time: 1 min

Expreimental replicate 3

	TSB	1X PBS	Synepure	Treatment TSB
	16	17	6	15
	14	18	7	19
	14	16	6	14
	5	5	3	5
	5	5	3	5
	5	5	3	5
	10	10	10	10
	10	10	10	10
	10	10	10	10
	10	10	10	10
	10	10	10	10
	10	10	10	10

	TSB	1X PBS	Synepure		Untreated
	1.60E+08	1.70E+08	6.00E+05		1.50E+08
	1.40E+08	1.80E+08	7.00E+05		1.90E+08
	1.40E+08	1.60E+08	6.00E+05		1.40E+08
**Avg**	**1.47E+08**	**1.70E+08**	**6.33E+05**	**Avg**	**1.60E+08**
STd dev				STd dev	

time: 3 min			Treatment time: 5 min				Treatment
**1X PBS**	**Synepure**		**TSB**	**1X PBS**	**Synepure**		**TSB**
17	5		16	14	3		17
14	7		15	13	4		15
14	9		11	15	2		19
5	2		5	5	0		5
5	2		5	5	0		5
5	2		5	5	0		5
10	10		10	10	10		10
10	10		10	10	10		10
10	10		10	10	10		10
10	10		10	10	10		10
10	10		10	10	10		10
10	10		10	10	10		10

**1X PBS**	**Catasyn**		**Untreated**	**1X PBS**	**Catasyn**		**Untreated**
1.70E+08	5.00E+04		1.60E+08	1.40E+08	3.00E+02		1.70E+08
1.40E+08	7.00E+04		1.50E+08	1.30E+08	4.00E+02		1.50E+08
1.40E+08	9.00E+04		1.10E+08	1.50E+08	2.00E+02		1.90E+08
**1.50E+08**	**7.00E+04**	**Avg**	**1.40E+08**	**1.40E+08**	**3.00E+02**	**Avg**	**1.70E+08**
		STd dev				STd dev	

time: 10 min

1X PBS	Synepure
15	2
14	5
12	4
5	0
5	0
5	0
10	10
10	10
10	10
10	10
10	10
10	10

1X PBS	Catasyn
1.50E+08	2.00E+02
1.40E+08	5.00E+02
1.20E+08	4.00E+02
**1.37E+08**	**3.67E+02**

Dr. Rubin Paper- Additional data
Catasyn (Lot 018-16; Exp 11/30/2019)
Date : 7/1/2019 Treatment time: 10 min

TSB	1X PBS	Catasyn
13	13	15
14	12	14
17	15	15
5	5	1
5	5	1
5	5	1
10	10	10
10	10	10
10	10	10
10	10	10
10	10	10
10	10	10

	**TSB**	**1X PBS**	**Catasyn**	
	1.30E+08	1.30E+08	1.50E+04	
	1.40E+08	1.20E+08	1.40E+04	
	1.70E+08	1.50E+08	1.50E+04	
**Avg**	**1.47E+08**	**1.33E+08**	**1.47E+04**	**Avg**
STd dev				STd dev

AV
STDEv

AV
STDEv

Treatment time: 30 min

TSB	1X PBS	Catasyn
12	12	13
15	11	14
14	13	15
5	5	0
5	5	0
5	5	0
10	10	10
10	10	10
10	10	10
10	10	10
10	10	10
10	10	10

Treatment time: 1 hr

TSB	1X PBS	Catasyn
17	16	0
19	15	0
18	17	0
5	5	0
5	5	0
5	5	0
10	10	10
10	10	10
10	10	10
10	10	10
10	10	10
10	10	10

**Untreated**	**1X PBS**	**Catasyn**		**Untreated**	**1X PBS**	**Catasyn**
1.20E+08	1.20E+08	1.30E+03		1.70E+08	1.60E+08	0.00E+00
1.50E+08	1.10E+08	1.40E+03		1.90E+08	1.50E+08	0.00E+00
1.40E+08	1.30E+08	1.50E+03		1.80E+08	1.70E+08	0.00E+00
**1.37E+08**	**1.20E+08**	**1.40E+03**	**Avg**	**1.80E+08**	**1.60E+08**	**0.00E+00**
			STd dev			

TSB

130000000	1.2E+08	1.7E+08	1.6E+08
140000000	1.5E+08	1.9E+08	1.4E+08
170000000	1.4E+08	1.8E+08	1.7E+08
**1.47E+08**	**1.37E+08**	**1.80E+08**	**1.57E+08**
18618986.7	13662601	8944272	13662601

1X PBS

130000000	1.2E+08	1.6E+08	1.6E+08
120000000	1.1E+08	1.5E+08	1.4E+08
150000000	1.3E+08	1.7E+08	1.7E+08
**1.33E+08**	**1.20E+08**	**1.60E+08**	**1.57E+08**
13662601	8944272	8944272	13662601

Treatment time: 24 hr

TSB	1X PBS	Catasyn
18	16	0
17	14	0
21	17	0
5	5	0
5	5	0
5	5	0
10	10	10
10	10	10
10	10	10
10	10	10
10	10	10
10	10	10

	**Untreated**	**1X PBS**	**Catasyn**
	1.80E+08	1.60E+08	0.00E+00
	1.70E+08	1.40E+08	0.00E+00
	2.10E+08	1.70E+08	0.00E+00
**Avg**	**1.87E+08**	**1.57E+08**	**0.00E+00**
STd dev			

Dr. Rubin Paper- Additional data
Catasyn (Lot 018-16; Exp 11/30/2019)

	Treatment time: 10 min			Treatment
Date : 7/1/2019				
Expreimental replicate 2	**TSB**	**1X PBS**	**Catasyn**	**TSB**
	16	16	14	16
	17	17	12	12
	13	15	16	15
	5	5	1	5
	5	5	1	5
	5	5	1	5
	10	10	10	10
	10	10	10	10
	10	10	10	10
	10	10	10	10
	10	10	10	10
	10	10	10	10

	**TSB**	**1X PBS**	**Catasyn**		**Untreated**
	1.60E+08	1.60E+08	1.40E+04		1.60E+08
	1.70E+08	1.70E+08	1.20E+04		1.20E+08
	1.30E+08	1.50E+08	1.60E+04		1.50E+08
**Avg**	**1.53E+08**	**1.60E+08**	**1.40E+04**	**Avg**	**1.43E+08**
STd dev				STd dev	

time: 30 min			Treatment time: 1 hr				Treatment
**1X PBS**	**Catasyn**		**TSB**	**1X PBS**	**Catasyn**		**TSB**
17	14		19	17	0		17
13	15		13	13	0		18
13	14		17	15	0		14
5	0		5	5	0		5
5	0		5	5	0		5
5	0		5	5	0		5
10	10		10	10	10		10
10	10		10	10	10		10
10	10		10	10	10		10
10	10		10	10	10		10
10	10		10	10	10		10
10	10		10	10	10		10

**1X PBS**	**Catasyn**		**Untreated**	**1X PBS**	**Catasyn**		**Untreated**
1.70E+08	1.40E+03		1.90E+08	1.70E+08	0.00E+00		1.40E+08
1.30E+08	1.50E+03		1.30E+08	1.30E+08	0.00E+00		1.50E+08
1.30E+08	1.40E+03		1.70E+08	1.50E+08	0.00E+00		1.20E+08
**1.43E+08**	**1.43E+03**	**Avg**	**1.63E+08**	**1.50E+08**	**0.00E+00**	**Avg**	**1.37E+08**
		STd dev				STd dev	

time: 24 hr

1X PBS	Catasyn
14	0
15	0
12	0
5	0
5	0
5	0
10	10
10	10
10	10
10	10
10	10
10	10

1X PBS	Catasyn
1.70E+08	0.00E+00
1.80E+08	0.00E+00
1.40E+08	0.00E+00
**1.63E+08**	**0.00E+00**

Dr. Rubin Paper- Additional data

Catasyn (Lot 018-16; Exp 11/30/2019)

Date : 7/1/2019	Treatment time: 10 min			Treatment
Expreimental replicate 3	**TSB**	**1X PBS**	**Catasyn**	**TSB**
	13	13	11	14
	14	16	10	16
	16	17	12	15
	5	5	1	5
	5	5	1	5
	5	5	1	5
	10	10	10	10
	10	10	10	10
	10	10	10	10
	10	10	10	10
	10	10	10	10
	10	10	10	10

	**TSB**	**1X PBS**	**Catasyn**		**Untreated**
	1.30E+08	1.30E+08	1.10E+04		1.40E+08
	1.40E+08	1.60E+08	1.00E+04		1.60E+08
	1.60E+08	1.70E+08	1.20E+04		1.50E+08
**Avg**	**1.43E+08**	**1.53E+08**	**1.10E+04**	**Avg**	**1.50E+08**
STd dev				STd dev	

time: 30 min			Treatment time: 1 hr				Treatment
**1X PBS**	**Catasyn**		**TSB**	**1X PBS**	**Catasyn**		**TSB**
13	14		17	16	0		17
14	13		13	15	0		16
15	13		12	12	0		19
5	0		5	5	0		5
5	0		5	5	0		5
5	0		5	5	0		5
10	10		10	10	10		10
10	10		10	10	10		10
10	10		10	10	10		10
10	10		10	10	10		10
10	10		10	10	10		10
10	10		10	10	10		10

**1X PBS**	**Catasyn**		**Untreated**	**1X PBS**	**Catasyn**		**Untreated**
1.30E+08	1.40E+03		1.70E+08	1.60E+08	0.00E+00		1.70E+08
1.40E+08	1.30E+03		1.30E+08	1.50E+08	0.00E+00		1.60E+08
1.50E+08	1.30E+03		1.20E+08	1.20E+08	0.00E+00		1.90E+08
**1.40E+08**	**1.33E+03**	**Avg**	**1.40E+08**	**1.43E+08**	**0.00E+00**	**Avg**	**1.73E+08**
		STd dev				STd dev	

time: 24 hr

1X PBS	Catasyn
19	0
17	0
14	0
5	0
5	0
5	0
10	10
10	10
10	10
10	10
10	10
10	10

1X PBS	Catasyn
1.90E+08	0.00E+00
1.70E+08	0.00E+00
1.40E+08	0.00E+00
**1.67E+08**	**0.00E+00**

Data generated for Hartman
Date: 7/1/2019
Wound rinse used:
synpure wound cleanser (Lot: 1701-19-028, Exp: 05/29/2022)

Anasep antimicrobial skin and wound cleanser (NDC 67180-400-16, Lot number: TL2915C, Exp: 2019-12-28)

Vashe skin- wound- burn cleansing solution (Lot : 16490, Exp: 08-2020)
Treatmnet time: 1 min

TSB	1X PBS	Synepure
19	17	10
17	16	9
15	14	10
5	5	1
5	5	1
5	5	1
10	10	10
10	10	10
10	10	10
10	10	10
10	10	10
10	10	10

	**TSB**	**1X PBS**	**Synepure**
	1.90E+08	1.70E+08	1.00E+04
	1.70E+08	1.60E+08	9.00E+03
	1.50E+08	1.40E+08	1.00E+04
**Avg**	**1.70E+08**	**1.57E+08**	**9.67E+03**
STd dev			
			9.44E+03

**Synepure**	**Synepure**	**TSB**	**1X PBS**	**Vashe**	**Vashe**	**Vashe**
11	10	17	15	11	13	12
10	9	16	13	14	14	11
8	8	18	16	12	13	10
1	1	5	5	3	3	3
1	1	5	5	3	3	3
1	1	5	5	3	3	3
10	10	10	10	10	10	10
10	10	10	10	10	10	10
10	10	10	10	10	10	10
10	10	10	10	10	10	10
10	10	10	10	10	10	10
10	10	10	10	10	10	10

**Synepure**	**Synepure**		**TSB**	**1X PBS**	**Vashe**	**Vashe**	**Vashe**
1.10E+04	1.00E+04		1.70E+08	1.50E+08	1.10E+06	1.30E+06	1.20E+06
1.00E+04	9.00E+03		1.60E+08	1.30E+08	1.40E+06	1.40E+06	1.10E+06
8.00E+03	8.00E+03		1.80E+08	1.60E+08	1.20E+06	1.30E+06	1.00E+06
**9.67E+03**	**9.00E+03**	**Avg**	**1.70E+08**	**1.47E+08**	**1.23E+06**	**1.33E+06**	**1.10E+06**
		STd dev					
					1.17E+06		

	TSB	1X PBS	Anasep	Anasep	Anasep
	18	17	12	10	12
	20	15	11	11	14
	17	12	10	9	11
	5	5	2	2	2
	5	5	2	2	2
	5	5	2	2	2
	10	10	10	10	10
	10	10	10	10	10
	10	10	10	10	10
	10	10	10	10	10
	10	10	10	10	10
	10	10	10	10	10

	TSB	1X PBS	Anasep	Anasep	Anasep
	1.80E+08	1.70E+08	1.20E+05	1.00E+05	1.20E+05
	2.00E+08	1.50E+08	1.10E+05	1.10E+05	1.40E+05
	1.70E+08	1.20E+08	1.00E+05	9.00E+04	1.10E+05
**Avg**	**1.83E+08**	**1.47E+08**	**1.10E+05**	**1.00E+05**	**1.23E+05**
STd dev					
			1.00E+05		

Data generated for Hartman
Date: 7/1/2019
Wound rinse used:
synpure wound cleanser (Lot: 1701-19-028, Exp: 05/29/2022)

Anasep antimicrobial skin and wound cleanser (NDC 67180-400-16, Lot number: TL2915C, Exp: 2019-12-28)

Vashe skin- wound- burn cleansing solution (Lot : 16490, Exp: 08-2020)
Treatmnet time: 3 min

**TSB**	**1X PBS**	**Synepure**
15	12	5
17	14	5
11	15	6
5	5	1
5	5	1
5	5	1
10	10	10
10	10	10
10	10	10
10	10	10
10	10	10
10	10	10

	**TSB**	**1X PBS**	**Synepure**
	1.50E+08	1.20E+08	5.00E+03
	1.70E+08	1.40E+08	5.00E+03
	1.10E+08	1.50E+08	6.00E+03
**Avg**	**1.43E+08**	**1.37E+08**	**5.33E+03**
STd dev			
			5.00E+03

Synepure	Synepure	TSB	1X PBS	Vashe	Vashe	Vashe
4	6	17	16	11	11	12
5	5	14	12	9	14	11
5	4	16	14	12	12	13
1	1	5	5	3	3	3
1	1	5	5	3	3	3
1	1	5	5	3	3	3
10	10	10	10	10	10	10
10	10	10	10	10	10	10
10	10	10	10	10	10	10
10	10	10	10	10	10	10
10	10	10	10	10	10	10
10	10	10	10	10	10	10

Synepure	Synepure		TSB	1X PBS	Vashe	Vashe	Vashe
4.00E+03	6.00E+03		1.70E+08	1.60E+08	1.10E+06	1.10E+06	1.20E+06
5.00E+03	5.00E+03		1.40E+08	1.20E+08	9.00E+05	1.40E+06	1.10E+06
5.00E+03	4.00E+03		1.60E+08	1.40E+08	1.20E+06	1.20E+06	1.30E+06
**4.67E+03**	**5.00E+03**	**Avg**	**1.57E+08**	**1.40E+08**	**1.07E+06**	**1.23E+06**	**1.20E+06**
		STd dev					
					1.23E+06		

TSB	1X PBS	Anasep	Anasep	Anasep
15	15	11	8	11
17	14	10	10	8
18	14	9	10	10
5	5	2	2	2
5	5	2	2	2
5	5	2	2	2
10	10	10	10	10
10	10	10	10	10
10	10	10	10	10
10	10	10	10	10
10	10	10	10	10
10	10	10	10	10

	**TSB**	**1X PBS**	**Anasep**	**Anasep**	**Anasep**
	1.50E+08	1.50E+08	1.10E+05	8.00E+04	1.10E+05
	1.70E+08	1.40E+08	1.00E+05	1.00E+05	8.00E+04
	1.80E+08	1.40E+08	9.00E+04	1.00E+05	1.00E+05
**Avg**	**1.67E+08**	**1.43E+08**	**1.00E+05**	**9.33E+04**	**9.67E+04**
STd dev					
			9.67E+04		

Data generated for Hartman
Date: 7/1/2019
Wound rinse used:
synpure wound cleanser (Lot: 1701-19-028, Exp: 05/29/2022)

Anasep antimicrobial skin and wound cleanser (NDC 67180-400-16, Lot number: TL2915C, Exp: 2019-12-28)

Vashe skin- wound- burn cleansing solution (Lot : 16490, Exp: 08-2020)
Treatmnet time: 5 min

TSB	1X PBS	Synepure
17	12	5
15	17	6
14	15	4
5	5	0
5	5	0
5	5	0
10	10	10
10	10	10
10	10	10
10	10	10
10	10	10
10	10	10

	TSB	1X PBS	Synepure
	1.70E+08	1.20E+08	5.00E+02
	1.50E+08	1.70E+08	6.00E+02
	1.40E+08	1.50E+08	4.00E+02
**Avg**	**1.53E+08**	**1.47E+08**	**5.00E+02**
STd dev			
			4.33E+02

Synepure	Synepure	TSB	1X PBS	Vashe	Vashe	Vashe
4	6	16	15	13	10	11
2	3	18	17	9	12	10
5	4	17	16	10	9	9
0	0	5	5	2	2	2
0	0	5	5	2	2	2
0	0	5	5	2	2	2
10	10	10	10	10	10	10
10	10	10	10	10	10	10
10	10	10	10	10	10	10
10	10	10	10	10	10	10
10	10	10	10	10	10	10
10	10	10	10	10	10	10

Synepure	Synepure		TSB	1X PBS	Vashe	Vashe	Vashe
4.00E+02	6.00E+02		1.60E+08	1.50E+08	1.30E+05	1.00E+05	1.10E+05
2.00E+02	3.00E+02		1.80E+08	1.70E+08	9.00E+04	1.20E+05	1.00E+05
5.00E+02	4.00E+02		1.70E+08	1.60E+08	1.00E+05	9.00E+04	9.00E+04
**3.67E+02**	**4.33E+02**	**Avg**	**1.70E+08**	**1.60E+08**	**1.07E+05**	**1.03E+05**	**1.00E+05**
		STd dev					
					9.33E+04		

	TSB	1X PBS	Anasep	Anasep	Anasep
	15	18	11	10	11
	13	17	12	10	10
	17	16	9	12	10
	5	5	1	1	1
	5	5	1	1	1
	5	5	1	1	1
	10	10	10	10	10
	10	10	10	10	10
	10	10	10	10	10
	10	10	10	10	10
	10	10	10	10	10
	10	10	10	10	10

	TSB	1X PBS	Anasep	Anasep	Anasep
	1.50E+08	1.80E+08	1.10E+04	1.00E+04	1.10E+04
	1.30E+08	1.70E+08	1.20E+04	1.00E+04	1.00E+04
	1.70E+08	1.60E+08	9.00E+03	1.20E+04	1.00E+04
**Avg**	**1.50E+08**	**1.70E+08**	**1.07E+04**	**1.07E+04**	**1.03E+04**
STd dev					
			1.03E+04		

Data generated for Hartman
Date: 7/1/2019
Wound rinse used:
synpure wound cleanser (Lot: 1701-19-028, Exp: 05/29/2022)

Anasep antimicrobial skin and wound cleanser (NDC 67180-400-16, Lot number: TL2915C, Exp: 2019-12-28)

Vashe skin- wound- burn cleansing solution (Lot : 16490, Exp: 08-2020)
Treatmnet time: 10 min

TSB	1X PBS	Synepure
17	16	2
15	17	4
14	16	3
5	5	0
5	5	0
5	5	0
10	10	10
10	10	10
10	10	10
10	10	10
10	10	10
10	10	10

	TSB	1X PBS	Synepure
	1.70E+08	1.60E+08	2.00E+02
	1.50E+08	1.70E+08	4.00E+02
	1.40E+08	1.60E+08	3.00E+02
**Avg**	**1.53E+08**	**1.63E+08**	**3.00E+02**
STd dev			
			2.33E+02

Synepure	Synepure	TSB	1X PBS	Vashe	Vashe	Vashe
3	4	16	12	9	8	8
3	2	15	15	8	9	8
2	2	15	14	8	7	7
0	0	5	5	1	1	1
0	0	5	5	1	1	1
0	0	5	5	1	1	1
10	10	10	10	10	10	10
10	10	10	10	10	10	10
10	10	10	10	10	10	10
10	10	10	10	10	10	10
10	10	10	10	10	10	10
10	10	10	10	10	10	10

Synepure	Synepure		TSB	1X PBS	Vashe	Vashe	Vashe
3.00E+02	4.00E+02		1.60E+08	1.20E+08	9.00E+03	8.00E+03	8.00E+03
3.00E+02	2.00E+02		1.50E+08	1.50E+08	8.00E+03	9.00E+03	8.00E+03
2.00E+02	2.00E+02		1.50E+08	1.40E+08	8.00E+03	7.00E+03	7.00E+03
**2.67E+02**	**2.67E+02**	**Avg**	**1.53E+08**	**1.37E+08**	**8.33E+03**	**8.00E+03**	**7.67E+03**
		STd dev					
					7.33E+03		

TSB	1X PBS	Anasep	Anasep	Anasep
15	16	7	8	7
15	14	8	6	6
17	15	6	6	8
5	5	1	1	1
5	5	1	1	1
5	5	1	1	1
10	10	10	10	10
10	10	10	10	10
10	10	10	10	10
10	10	10	10	10
10	10	10	10	10
10	10	10	10	10

	TSB	1X PBS	Anasep	Anasep	Anasep
	1.50E+08	1.60E+08	7.00E+03	8.00E+03	7.00E+03
	1.50E+08	1.40E+08	8.00E+03	6.00E+03	6.00E+03
	1.70E+08	1.50E+08	6.00E+03	6.00E+03	8.00E+03
**Avg**	**1.57E+08**	**1.50E+08**	**7.00E+03**	**6.67E+03**	**7.00E+03**
STd dev					
			6.67E+03		
