# Supplementary material for: Soluble chitosan derivative treats wound infections and promotes wound healing in a novel MRSA-infected porcine partial-thickness burn wound model
Source: PLoS One. 2022 Oct 14;17(10):e0274455. doi: 10.1371/journal.pone.0274455 (PMC9565743; doi:10.1371/journal.pone.0274455)
Supplement: Attachment — Submitted filename: Comments and suggestions for Authors - PONE-D-22-02846.docx [file pone.0274455.s005.docx]

Dear authors,

Thank you for submitting your manuscript. However, I am returning it to you without further review in order not to delay its eventual publication. Therefore, I do not think that the manuscript is suitable to the journal and rejected due to below-following points:

1. The abstract should be rewritten to focus on the aim of this study. This study does not show the main problem to be solved.
2. The novelty of this study must be highlighted. MRSA-infected porcine PTBW model and materials prepared from chitosan derivative were researched popularly and applied for many medicines applications.
3. In the line 341 and 342, the authors said, “the model was designed to reduce cost”. authors should explain more clearly. How does the model reduce cost from this study?
4. Line 347 and 348, the authors said, “this model can be used to save time and reduce animal resources”. Another animal resources will be chosen for different purposes.
5. The figures in the manuscript are not clear and the quality of images should be improved. Furthermore, figure S1 is not clear to read.
6. Language needs substantial improvement.
7. There are many spelling mistakes in the manuscript. Authors should replace “was” word by “were” word in line 119; “scars were inferior” by “scars was inferior” line 291, “treatment lead to” by “treatment led to” in line 303; “dressing change” by “dressing changes” in line 312; “bacteria present in” by “bacteria presents in” in line 354.

Thank you, once again, for submitting the manuscript.

Dr. Thi-Hiep NGUYEN
